# A novel disulfidptosis-related lncRNA signature to predict prognosis and immune response of cervical cancer

**DOI:** 10.1097/MD.0000000000046023

**Published:** 2025-11-21

**Authors:** Hu Zhao, Yilin Guo, Lu Wang, Rui Li, Yingmei Wang

**Affiliations:** a Department of Gynecology and Obstetrics, Tianjin Medical University General Hospital, Tianjin, China; b Department of Gynecology and Obstetrics, The Second Affiliated Hospital of Zhengzhou University, Zhengzhou, China.

**Keywords:** cervical cancer, disulfidptosis, immunotherapy, lncRNAs, prognosis

## Abstract

Disulfidptosis, a new identified form of regulated cell death, has been implicated in cancer. However, the mechanisms through which disulfidptosis-related long noncoding RNAs (lncRNAs) predict prognosis in cervical cancer (CC) remain unclear. Here, we identified disulfidptosis-related genes and lncRNAs in the cancer genome atlas database. Least absolute shrinkage and selection operator and Cox regression analyses were used to construct a prognostic risk signature based on optimal disulfidptosis-related lncRNAs. The prognostic performance of the signature was evaluated using Kaplan–Meier survival analysis and receiver operating characteristic curves. Correlation between the risk signature, gene mutation landscape, tumor immune microenvironment, and immunotherapy or chemotherapy sensitivity was determined. Additionally, the expression levels of disulfidptosis-related lncRNAs in CC were validated by quantitative PCR. A total of 403 disulfidptosis-related lncRNAs were identified, among which 9 disulfidptosis-related lncRNAs were used to construct a prognostic risk signature that classified patients with CC into high-risk and low-risk groups. Kaplan–Meier, receiver operating characteristic curves, and the concordance index demonstrated that the risk signature exhibited good sensitivity and specificity. The low-risk group exhibited improved survival outcomes and increased sensitivity to immunotherapy, whereas the high-risk group showed heightened sensitivity to to bexarotene, bicalutamide, embelin, FH535, and pazopanib. Quantitative PCR results indicated that ILF3-DT and PPP1R14B-AS1 were downregulated in CC tissues, whereas RUSC1-AS1 was upregulated. In conclusion, we developed a novel prognostic risk signature based on 9 disulfidptosis-related lncRNAs, which may serve as an independent predictor of immunotherapy response and chemotherapy sensitivity in CC.

## 1. Introduction

Cervical cancer (CC) ranks 4th in terms of incidence and mortality among women, with an estimated 661,021 new cases and 348,189 deaths annually.^[1]^ Nearly all patients diagnosed CC are associated with high-risk human papillomavirus infection.^[2]^ Although the implementation of early screening programs and human papillomavirus vaccination has improved early diagnosis and treatment in developing countries, metastatic and recurrent CC continues to pose significant clinical challenges.^[3]^ Current treatment strategies, including surgery, chemotherapy, and radiotherapy, have shown limited efficacy in improving outcomes for patients with advanced or recurrent CC.^[4]^ Therefore, there is an urgent need to investigate the molecular mechanisms underlying CC and to identify reliable prognostic biomarkers to guide individualized treatment.

Based on their morphology, biochemistry, and function, modes of cell death can be categorized into accidental and regulated cell death (RCD).^[5]^ Advances in tumor cell biology and cancer therapeutics have led to the identification of multiple RCD subtypes, including necroptosis, pyroptosis, ferroptosis, and cuproptosis.^[6,7]^ Disulfidptosis, a newly recognized form of RCD induced by disulfide stress, has garnered significant attention in tumor research.^[8]^ Current findings indicate that the accumulation of disulfide materials alters the conformation of cytoskeletal proteins, leading to the collapse of the actin network and cell death in glucose-deficient cancer cells with high SLC7A11 expression.^[9]^ These findings suggest that disulfidptosis may represent a promising therapeutic target for cancer treatment^[10]^; however, its regulatory role in CC has not been systematically investigated.

Long noncoding RNAs (lncRNAs), defined as transcripts longer than 200 nucleotides, play crucial roles in various biological processes such as transcriptional and posttranscriptional regulation, chromatin remodeling, and epigenetic modulation.^[11]^ Accumulating evidence has revealed the dysregulation of lncRNAs in CC, enhancing our understanding of its biological behavior and offering new treatment prospects.^[12]^ Recent studies have focused on developing prognostic prediction models based on various types of lncRNAs. For example, PANoptosis-related lncRNA signatures constructed via least absolute shrinkage and selection operator (LASSO) regression have been established for lung adenocarcinoma^[13]^ and pancreatic adenocarcinoma,^[14]^ with validation through Kaplan–Meier, Cox, and receiver operating characteristic (ROC) analyses. Nevertheless, the prognostic value of disulfidptosis-related lncRNAs in CC remains unexplored. Therefore, our study aimed to construct an innovative prognostic signature based on 9 identified disulfidptosis-related lncRNAs, and evaluate its utility in predicting overall survival (OS), progression-free survival (PFS), and immune infiltration in patients with CC, thereby providing new insights for precision medicine.

## 2. Materials and methods

### 2.1. Data collection and processing

The RNA-seq transcriptome profiles of 304 CC and 3 normal cervical samples were downloaded from the cancer genome atlas (TCGA) database (https://portal.gdc.cancer.gov/). Clinical information, including age, pathological grade, clinical stage, tumor node metastasis stage, survival status, and survival time, was also retrieved. We applied the ComBat method from the R package “sva” for standardization. A total of 304 CC patients were finally enrolled and randomly divided into a training cohort (n = 183) and a validation cohort (n = 121) at a 6:4 ratio^[15]^ using the R “caret” package, as shown in Table 1.

**Table 1:** Characteristic of cervical cancer patients involved in the study.

Variable	TCGA cohort (n = 304)	Validation cohort (n = 121)	Training cohort (n = 183)	*P* value
Age				
≤65	269 (88.49%)	106 (87.6%)	163 (89.07%)	.8345
>65	35 (11.51%)	15 (12.4%)	20 (10.93%)	
Grade				
G1	18 (5.92%)	10 (8.26%)	8 (4.37%)	.3223
G2	135 (44.41%)	54 (44.63%)	81 (44.26%)	
G3	119 (39.14%)	44 (36.36%)	75 (40.98%)	
Unknown	32 (10.53%)	13 (10.74%)	19 (10.38%)	
Stage				
Stage I	162 (53.29%)	69 (57.02%)	93 (50.82%)	.3570
Stage II	69 (22.7%)	29 (23.97%)	40 (21.86%)	
Stage III	45 (14.8%)	16 (13.22%)	29 (15.85%)	
Stage IV	21 (6.91%)	5 (4.13%)	16 (8.74%)	
Unknown	7 (2.3%)	2 (1.65%)	5 (2.73%)	
T stage				
T1	141 (46.38%)	55 (45.45%)	86 (46.99%)	.3759
T2	71 (23.36%)	28 (23.14%)	43 (23.5%)	
T3	20 (6.58%)	4 (3.31%)	16 (8.74%)	
T4	10 (3.29%)	3 (2.48%)	7 (3.83%)	
Unknown	62 (20.39%)	31 (25.62%)	31 (16.94%)	
M stage				
M0	116 (38.16%)	39 (32.23%)	77 (42.08%)	.0643
M1	10 (3.29%)	0 (0%)	10 (5.46%)	
Unknown	178 (58.55%)	82 (67.77%)	96 (52.46%)	
N stage				
N0	133 (43.75%)	50 (41.32%)	83 (45.36%)	.7056
N1	60 (19.74%)	25 (20.66%)	35 (19.13%)	
Unknown	111 (36.51%)	46 (38.02%)	65 (35.52%)	

TCGA = the cancer genome atlas.

### 2.2. Identification of disulfidptosis-related genes and lncRNAs

Ten disulfidptosis-related genes (*GYS1*, *NDUFS1*, *OXSM*, *LRPPRC*, *NDUFA11*, *NUBPL*, *NCKAP1*, *RPNA1*, *SLC3A2*, and *SLC7A11*) were selected based on a previous study by Liu et al.^[8]^ The “limma” package was used to identify differential expression of disulfidptosis-related genes between CC and normal cervical samples. A correlation analysis was performed to identify disulfidptosis-related lncRNAs with a threshold (cor > 0.3 and *P* < .001).

### 2.3. Construction and validation of disulfidptosis-related prognostic lncRNA risk signature

To verify the disulfidptosis-related prognostic lncRNAs, univariate Cox regression analysis was applied to survival data using the “survival” R package. Moreover, LASSO regression analysis was conducted to optimize disulfidptosis-related prognostic lncRNAs and prevent data overfitting. Multivariate stepwise Cox analysis was used to construct a prognostic risk signature based on the optimal disulfidptosis-related lncRNAs. Based on the median risk score, patients with CC were divided into high- and low-risk groups in the training and validation cohorts. Kaplan–Meier analysis was performed to compare the OS and PFS between the 2 groups. The 1, 3, and 5-year survival rates of the ROC and concordance index (C-index) curves were calculated to estimate the performance of the risk signature. Independent risk factors were explored using univariate and multivariate Cox regression analyses to determine the prognostic value of the risk signature.

### 2.4. Establishment of nomogram and analysis of clinicopathological subgroups

A nomogram was established according to the risk score in the risk signature and other clinicopathological features, including grade, stage, and age. Calibration curve analysis and the C-index were plotted to verify the stability and accuracy of the nomogram. Subgroup analyses based on clinicopathological features were performed using Kaplan–Meier survival curves.

### 2.5. Principal component analysis (PCA) and functional enrichment analysis

To visualize the spatial distribution of high-risk and low-risk groups, PCA was used to classify the expression patterns of disulfidptosis-related lncRNAs and genes of CC using the “scatterplot3d” package. Gene ontology analysis, which consisted of 3 domains: biological process, cellular component, and molecular function was explored between high-risk and low-risk groups based on the “org.Hs.e.g..db,” “enrichplot,” and “clusterProfiler” package. Gene set enrichment analysis and gene set variation analysis were performed to identify the significantly enriched pathways in CC, with an adjusted *P* value < .05.

### 2.6. Estimation of immune cell infiltration and immunotherapy

The cell-type identification by estimating relative subsets of RNA transcripts (CIBERSORT) and estimation of stromal and immune cells in malignant tumors using expression data (ESTIMATE) algorithms were used to assess immune cell infiltration and the proportion of components in the tumor microenvironment. XCELL and quantification of the tumor immune contexture from human RNA-seq data algorithms were used to investigate the correlation between the prognostic risk signature and immune cell infiltration.

Recently, immunotherapy has been gradually applied for patients with CC. Consequently, we evaluated the differential expression of immune-related genes, including immune checkpoints and human leukocyte antigen (HLA), in high- and low-risk groups of CC. The tumor immune dysfunction and exclusion (TIDE) score (http://tide.dfci.harvard.edu) and immunophenoscore (IPS) (https://tcia.at/home) were used to predict the response to immunotherapy of patients with CC between the 2 groups.

### 2.7. Landscape of gene mutation and tumor somatic mutation

Somatic mutation data were downloaded from TCGA and integrated using the Perl script (version 5.30.0). We used “maftools” R package to create the mutational waterfall plot and calculate the tumor mutation burden (TMB) of each patient with CC. TMB, defined as the number of somatic mutations per million bases in a coding region, is a predictive indicator of the efficacy of immunotherapy. According to the median TMB value, we divided the patients with CC into high-TMB and low-TMB groups. A Kaplan–Meier curve was plotted to compare survival differences between the 2 groups.

### 2.8. Drug sensitivity analysis

The half-maximal inhibitory concentration values of various antitumor drugs were calculated using the “pRRophetic” package between the high-risk and low-risk groups, in order to assess the role of in the prognostic risk signature in forecasting response to therapy. Differences in drug sensitivity between risk groups were compared using the Wilcoxon signed-rank test.

### 2.9. RNA extraction and real-time quantitative PCR (qPCR)

Five pairs of CC and adjacent normal samples were acquired from patients at The Second Affiliated Hospital of Zhengzhou University between 2021 to 2022 to verify the expression levels of 3 disulfidptosis-related lncRNAs in the prognostic risk signature using real-time qPCR. Total RNA was extracted from the tissue samples using TRIzol reagent. Reverse transcription was performed using a HiScript II 1st Strand cDNA Synthesis Kit (Nanjing Novozan Biotechnology Co., Ltd.). qPCR was carried out using 2 × Synergetic Binding Reagent Green qPCR Master Mix (Wuhan Servicebio Technology Co., Ltd.). With glyceraldehyde 3-phosphate dehydrogenase as the internal reference, the expression of lncRNAs was analyzed and quantified by the 2−^ΔΔCt^ method. The primer sequences are shown in Table 2. This study was approved by the ethical committee of The Second Affiliated Hospital of Zhengzhou University (Approval No. 2021040).

**Table 2:** Primer sequences of lncRNAs in the real-time PCR.

lncRNAs	Primer sequences
PPP1R14B-AS1	Forward: 5′-TGCTACCAGGCTTGAACAG-3′
	Reverse: 5′-CAGGCACAGAGGAAGACAT-3′
RUSC1-AS1	Forward: 5′-TGCATTTGTTGTCCTGGATG-3′
	Reverse: 5′-GCTGGTTTCAGGGTACAGGA-3′
ILF3-DT	Forward: 5′-AATATCTCACTAGAGGGCTCACCG-3′
	Reverse: 5′-CCTGGAAGCATTCAAGAACACC-3′
GAPDH	Forward: 5′-ACCCACTCCTCCACCTTTGACG-3′
	Reverse: 5′-TCTCTTCCTCTTGTGCTCTTG-3′

GAPDH = glyceraldehyde 3-phosphate dehydrogenase, lncRNAs = long noncoding RNAs.

### 2.10. Statistical analysis

Data processing and statistical analyses were performed using R software (version 4.1.2) and Perl (version 5.30.0). Group comparisons were conducted using the Wilcoxon test or Chi-square test, as appropriate. Correlations were assessed using Spearman correlation coefficient. A *P*-value < .05 was considered statistically significant.

## 3. Results

### 3.1. Correlation and prognostic value of disulfidptosis-related genes

**Figure 1.**
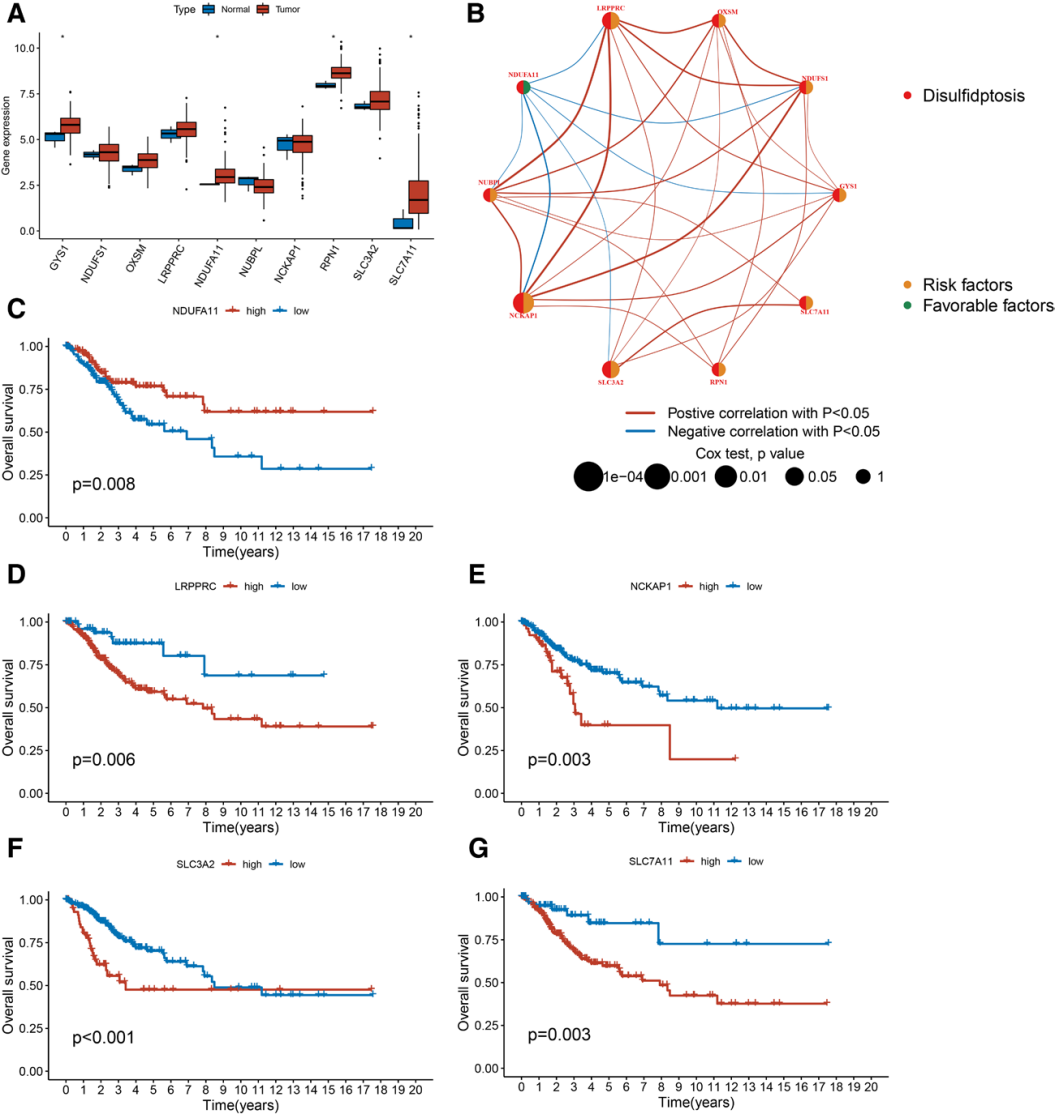
Analysis of disulfidptosis-related genes in CC. (A) The expression of 10 disulfidptosis-related genes in CC and normal cervical samples. (B) Correlation analysis of 10 disulfidptosis-related genes in CC. (C) Kaplan–Meier survival curves of NDUFA11 in CC. (D) Kaplan–Meier survival curves of LRPPRC in CC. (E) Kaplan–Meier survival curves of NCKAP1 in CC. (F) Kaplan–Meier survival curves of SLC3A2 in CC. (G) Kaplan–Meier survival curves of SLC7A11 in CC. CC = cervical cancer.

The expression levels of 10 disulfidptosis-related genes were compared between 304 CC samples and 3 normal cervical samples. Our findings revealed that the expression levels of *GYS1*, *NDUFA11*, *RPN1*, and *SLC7A11* were significantly upregulated in CC samples (Fig. 1A). A spearman correlation analysis was performed to evaluate relationships among the 10 disulfidptosis-related genes (Fig. 1B). With the exception of *NDUFA11*, most genes exhibited a positive correlation. To investigate the impact on prognosis, we conducted a Kaplan–Meier analysis. The results demonstrated that patients with CC with high *NDUFA11* expression was associated with better OS (Fig. 1C), whereas high expression levels of *LRPPRC*, *NCKAP1*, *SLC3A2*, and *SLC7A11* predicted poorer OS (Fig. 1D–G).

### 3.2. Construction of a prognostic risk signature of disulfidptosis-related lncRNAs

**Figure 2.**
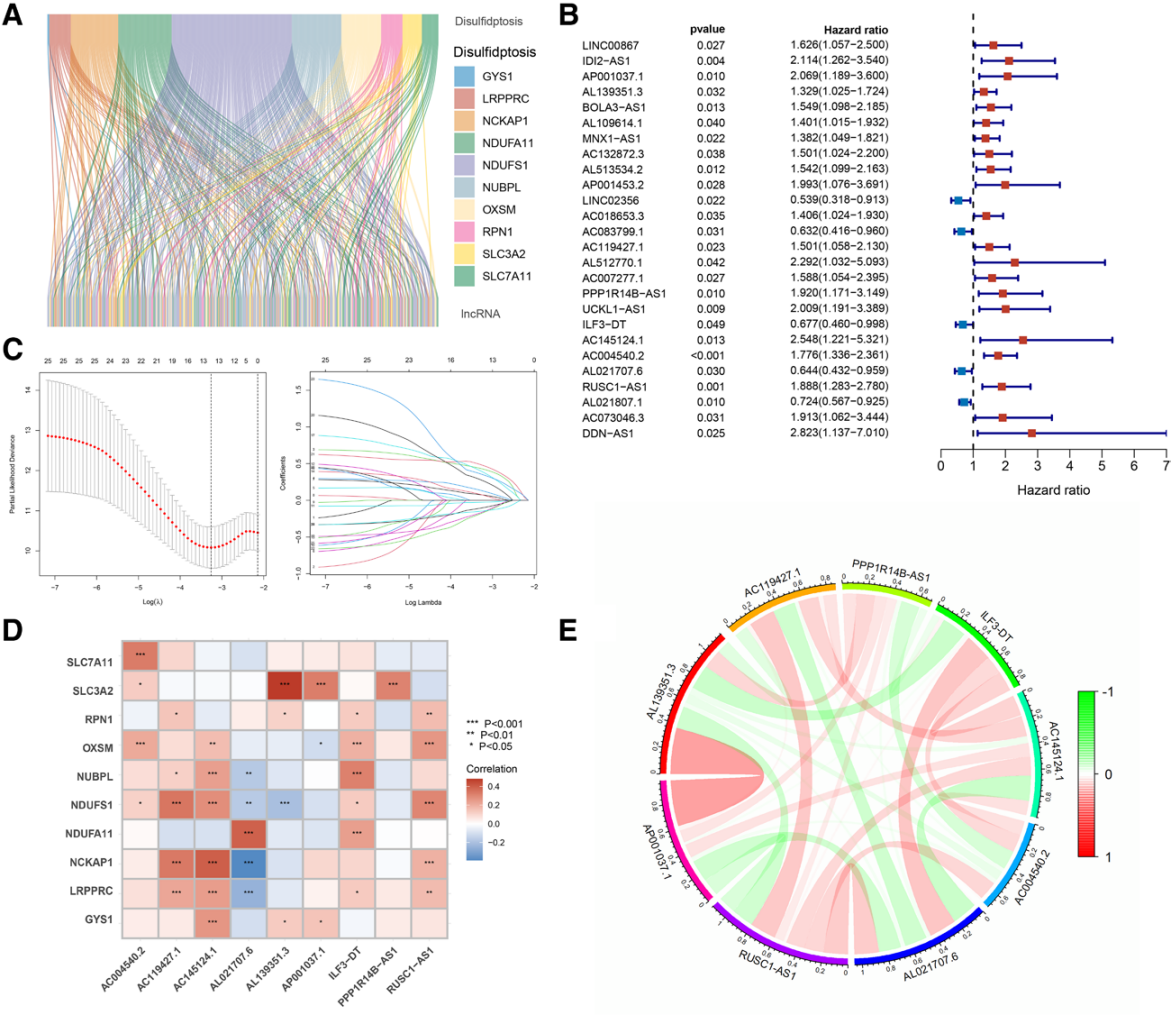
Screening of disulfidptosis-related lncRNAs and construction of a prognostic risk signature in CC. (A) Sankey diagram of the co-expression of cuproptosis-related genes and lncRNAs in CC. (B) Forest plot of disulfidptosis-related lncRNAs associated with survival in CC. (C) LASSO regression analysis for further screening. (D) Spearman correlation analysis of disulfidptosis-related genes and lncRNAs in a prognostic risk signature. Red represents positive correlation, and blue represents negative correlation. (E) Spearman correlation analysis of disulfidptosis-related lncRNAs in a prognostic risk signature. Red represents positive correlation, and green represents negative correlation. CC = cervical cancer, LASSO = least absolute shrinkage and selection operator, lncRNAs = long noncoding RNAs.

As shown in Figure 2A, a total of 403 disulfidptosis-related lncRNAs were identified, among which 393 were found to be upregulated and ten downregulated in CC samples (correlation coefficient > 0.3 and *P* < .001). A cohort of 304 patients with CC were randomly divided into a training cohort (n = 183) and a validation cohort (n = 121), as shown in Table 1. In the training cohort, univariate Cox regression analysis indicated that 26 disulfidptosis-related lncRNAs were significantly associated with survival, leading to the construction of a forest plot (Fig. 2B). Further screening with LASSO regression analysis identified 13 disulfidptosis-related lncRNAs (Fig. 2C). A prognostic risk signature comprising 9 disulfidptosis-associated lncRNAs was developed using multivariate Cox regression analysis. Subsequently, Spearman correlation analysis was performed to evaluate the correlation between the 9 disulfidptosis-related lncRNAs and 10 disulfidptosis-related genes (Fig. 2D). Additionally, the correlations among the expression levels of the 9 disulfidptosis-related lncRNAs was assessed (Fig. 2E).

**Figure 3.**
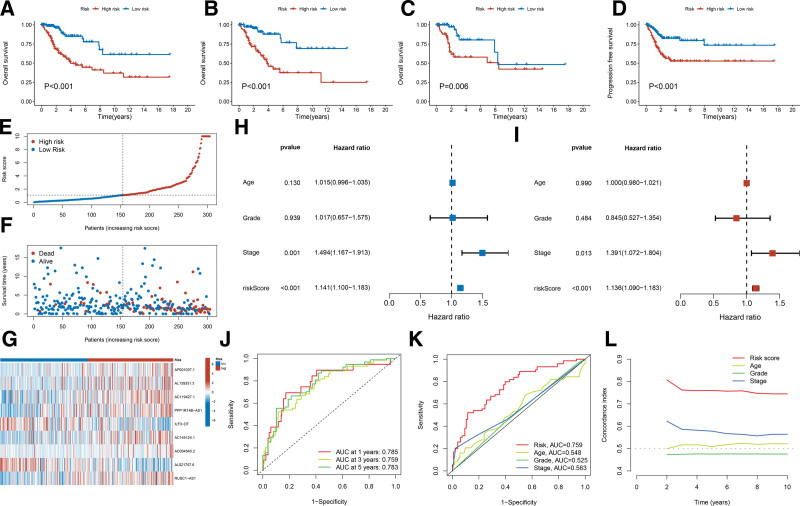
Evaluation of prediction ability of the prognostic risk signature in CC. (A) Kaplan–Meier survival curves on OS status in TCGA cohort. (B) Kaplan–Meier survival curves on OS status in training cohort. (C) Kaplan–Meier survival curves on OS status in validation cohort. (D) Kaplan–Meier survival curves on PFS status in TCGA cohort. (E) Risk score distribution of CC patients in TCGA cohort. (F) Survival status distribution of CC patients in TCGA cohort. (G) The heatmap of the expression levels of 9 disulfidptosis-related lncRNAs in TCGA cohort. (H) Univariate Cox regression analysis of the clinicopathological features and prognostic risk signature. (I) Multivariate Cox regression analysis of the clinicopathological features and prognostic risk signature. (J) ROC curve of 1-, 3-, and 5-years in TCGA cohort. (K) ROC curve of risk score and clinicopathological features in TCGA cohort. (L) The C-index curves of risk score and clinicopathological features at each time point. CC = cervical cancer, C-index = concordance index, lncRNAs = long noncoding RNAs, OS = overall survival, PFS = progression-free survival, ROC = receiver operating characteristic, TCGA = the cancer genome atlas.

Using the median risk score as the cutoff value in the training, validation, and TCGA cohorts (n = 304), patients with CC were categorized into low-risk and high-risk groups. Kaplan–Meier analysis demonstrated that the OS of the high-risk group was significantly lower than that of the low-risk group (Fig. 3A–C). Similarly, the PFS of the high-risk group was markedly shorter than that of the low-risk group (Fig. 3D). The risk score, survival status, and gene expression data for the training, validation, and TCGA cohorts are illustrated in Figure 3E–G and Figure S1A–F, Supplemental Digital Content, https://links.lww.com/MD/Q658. Furthermore, both univariate and multivariate Cox regression analyses indicated that clinical stage and risk score were in-dependent risk factors for CC (Fig. 3H–I). In the TCGA cohort, the ROC curve was utilized to evaluate the specificity and sensitivity of the risk prognostic signature. The areas under the receiver operating characteristic curves (AUC) for 1, 3, and 5-years were 0.786, 0.759, and 0.783, respectively (Fig. 3J). The AUC also demonstrated favorable results in the training and validation cohorts (Fig. S1G and I, Supplemental Digital Content, https://links.lww.com/MD/Q658). Moreover, the AUC and C-index associated with the risk score were superior to those for age, histological grade, and clinical stage (Fig. 3K–L, Fig. S1H and J, Supplemental Digital Content, https://links.lww.com/MD/Q658). These findings substantiate that the prognostic risk signature based on the 9 disulfidptosis-related lncRNAs possesses a robust capability to predict the survival of patients with CC.

### 3.3. Establishment of a nomogram

To assess the clinical applicability of the prognostic risk signature, we developed a nomogram integrating clinicopathological features and risk score to predict the 1-, 3-, and 5-year survival rates of 304 patients with CC (Fig. S2A, Supplemental Digital Content, https://links.lww.com/MD/Q658). The C-index of the nomogram was found to be 0.723, and the calibration curve demonstrated close alignment between the predicted and ideal curves, further demonstrating a high level of consistency between the predicted and actual outcomes (Fig. S2B, Supplemental Digital Content, https://links.lww.com/MD/Q658).

### 3.4. PCA and subgroup analysis of clinicopathological features

We employed PCA to visualize the distribution of CC patients between high-risk and low-risk groups across the total gene expression profiles, disulfidptosis-related gene expression profiles, disulfidptosis-related lncRNAs expression profiles, and 9 disulfidptosis-related lncRNAs derived from the prognostic risk signature. The results indicated that the 9 disulfidptosis-related lncRNAs effectively distinguished the distribution of the 304 patients with CC in the 2 subgroups (Fig. S3A–D, Supplemental Digital Content, https://links.lww.com/MD/Q658), further confirming the accuracy of the prognostic risk signature, which was corroborated by subgroup analyses that included variables such as age, histological grade, and clinical stage. Notably, both young patients (age ≤ 65 years) and elderly patients (age > 65 years), as well as those with low histological grades (G1–G2) and high histological grades (G3), and early clinical stages (I–II) versus advanced clinical stages (III–IV), demonstrated statistical significance as determined by Kaplan–Meier analysis (Fig. S2C–H, Supplemental Digital Content, https://links.lww.com/MD/Q658). These findings suggest that the prognostic risk signature serves as a robust tool for predicting CC survival across various clinicopathological subgroups categorized by age, histological grade, and clinical stage.

### 3.5. Functional enrichment analysis

To demonstrate the biological functions and signaling pathways between the high-risk and low-risk groups, we performed gene ontology enrichment analysis of differentially expressed genes (DEGs). The results revealed that the DEGs related to biological processes were predominantly involved in the regulation of angiogenesis, vasculature development, and cellular responses to biotic stimuli. In terms of cellular components, DEGs were associated with the collagen-containing extracellular matrix, T cell receptor complex, and laminin complex. Regarding molecular functions, DEGs exhibited significant enrichment in cytokine activity, growth factor receptors, extracellular matrix binding, and receptor ligand activity (Fig. S3E, Supplemental Digital Content, https://links.lww.com/MD/Q658). Furthermore, gene set variation analysis demonstrated a significant positive correlation between the risk score in the prognostic risk signature and multiple Kyoto encyclopedia of genes and genomes pathways, including WNT, VEGF, P53, NOTCH, and MAPK (Fig. S3F, Supplemental Digital Content, https://links.lww.com/MD/Q658). Gene set enrichment analysis revealed that the high-risk group was primarily enriched in TGF-β, extracellular matrix receptor interactions, and focal adhesion pathways. In contrast, the low-risk group exhibited enrichment in allograft rejection, oxidative phosphorylation, and ribosome pathways (Fig. S3G, Supplemental Digital Content, https://links.lww.com/MD/Q658).

### 3.6. Landscape of mutation profiles and survival analysis

To explore the landscape of somatic mutation profiles between high-risk and low-risk groups, somatic mutation data from the TCGA database were obtained. A waterfall plot illustrates the 5 genes (*TTN*, *PIK3CA*, *KMT2C*, *MUC16*, and *KMT2D*) with the highest mutation frequencies (Fig. S4A and B, Supplemental Digital Content, https://links.lww.com/MD/Q658). The somatic mutation rate in the low-risk group was 84.62%, slightly higher than that in the high-risk group (80.14 %); however, the difference was not statistically significant (*P* = .7).

Thus, TMB can serve as a biomarker for tumor prognosis and immunotherapy. We calculated the TMB for each patient with CC and found no significant difference between the high- and low-risk groups (*P* = .58, Fig. S4C, Supplemental Digital Content, https://links.lww.com/MD/Q658). However, patients with CC in the high-TMB group exhibited significantly better prognoses compared to those in the low-TMB group (Fig. S4D, Supplemental Digital Content, https://links.lww.com/MD/Q658). Moreover, the combination of TMB with a prognostic risk signature has clinical significance in the prognosis of CC. Notably, patients in the high-TMB and low-risk groups exhibited the best prognosis, whereas those in the low-TMB and high-risk groups had the lowest survival probability *(P* < .001; Fig. S4E, Supplemental Digital Content, https://links.lww.com/MD/Q658). In addition, further analysis revealed that the high-risk group showed a higher expression of proliferation-, DNA repair-, and angiogenesis-related genes (Fig. S4F, Supplemental Digital Content, https://links.lww.com/MD/Q658), which could potentially explain the worse prognosis observed in this group.

### 3.7. Characteristics of immune infiltration

**Figure 4.**
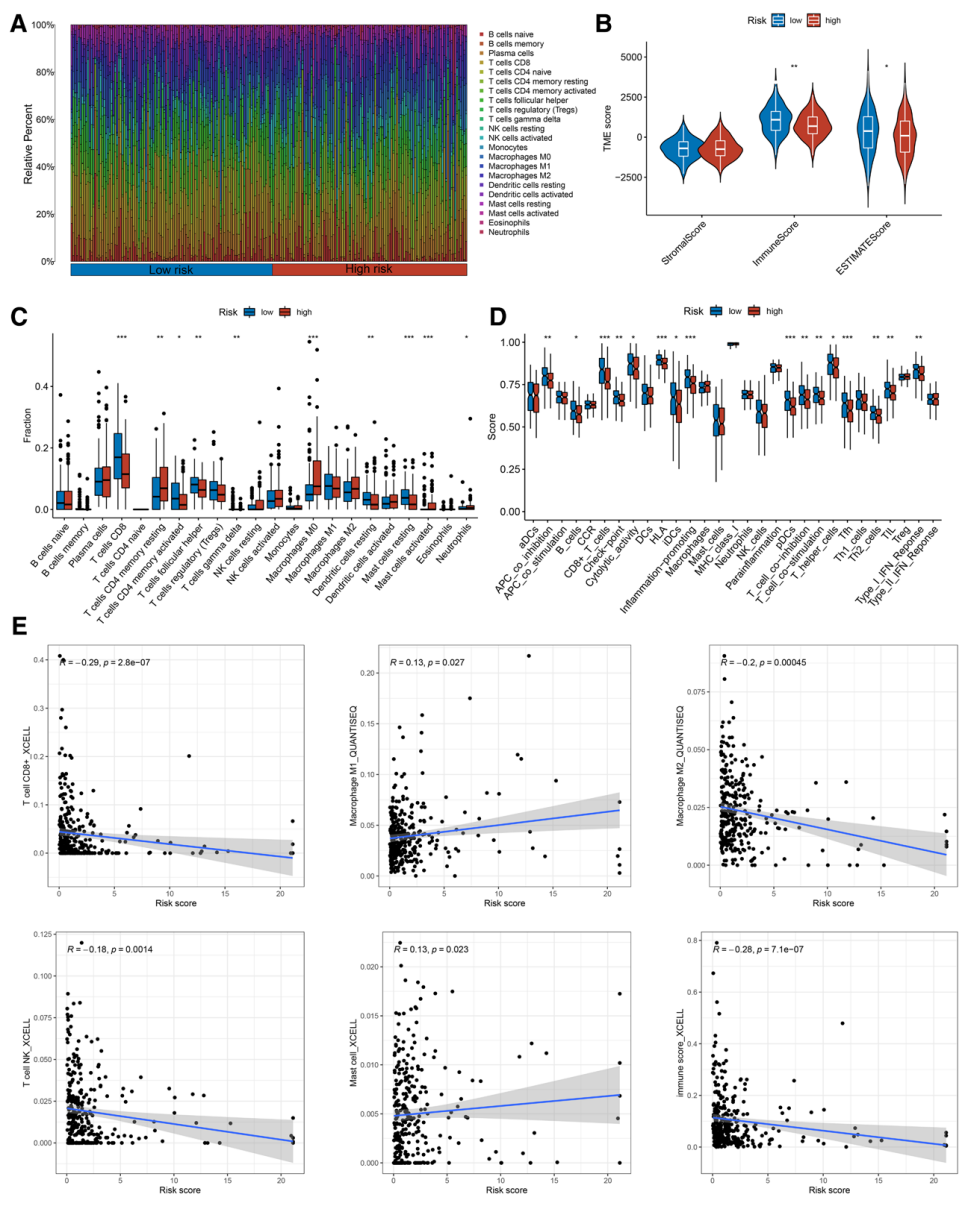
The characteristics of immune infiltration in the prognostic risk signature. (A) The CIBERSORT algorithm of immune cell infiltration panorama in CC. (B) The ESTIMATE algorithm of immune and stromal components in high-risk and low-risk groups. The asterisks represented the statistical *P* value (*P < .05; **P < .01). (C) The infiltration of 22 immune cell types in high-risk and low-risk groups. The asterisks represented the statistical *P* value (*P < .05; **P < .01; ***P < .001). (D) The difference of immune-related functions in high-risk and low-risk groups. The asterisks represented the statistical *P* value (*P < .05; **P < .01; ***P < .001). (E) Correlation analysis of risk score and immune cell infiltration. CC = cervical cancer, CIBERSORT = the cell-type identification by estimating relative subsets of RNA transcripts, ESTIMATE = estimation of stromal and immune cells in malignant tumors using expression data.

To investigate the impact of the tumor immune microenvironment on the occurrence, metastasis, and effectiveness of immunotherapy for CC, we investigated the differences between high- and low-risk groups. The CIBERSORT algorithm was employed to assess the characteristics of 22 immune cell infiltrates (Fig. 4A). The ESTIMATE algorithm was used to identify the immune and stromal components. Notably, the ImmuneScore and ESTIMATEScore of the low-risk group were significantly higher than those of the high-risk group (Fig. 4B). In the low-risk group, there was significant enrichment of CD8^+^ T cells, activated CD4^+^ memory T cells, follicular helper T cells, resting dendritic cells, and resting mast cells (Fig. 4C). Additionally, the scores for HLA, immune checkpoints, cytokines, and activated immune promotion were elevated in the low-risk group, suggesting a relationship with the immune activation phenotype (Fig. 4D). Conversely, the high-risk group showed significant enrichment of resting CD4^+^ memory T cells, M0 macrophages, activated mast cells, and neutrophils, indicating a potential association with immunosuppression (Fig. 4C). The XCELL and quantification of the tumor immune contexture from human RNA-seq data algorithms were used to assess the correlation between the risk score and immune cell infiltration. The findings revealed a negative correlation between the risk score and CD8^+^ T cell, M2 macrophage, natural killer cell, and immune scores (Fig. 4E). However, a positive correlation was observed between the risk score and M1 macrophages (Fig. 4E). These results provide a novel perspective on the tumor immune microenvironment in CC.

### 3.8. Sensitivity analysis of immunotherapy and chemotherapy

**Figure 5.**
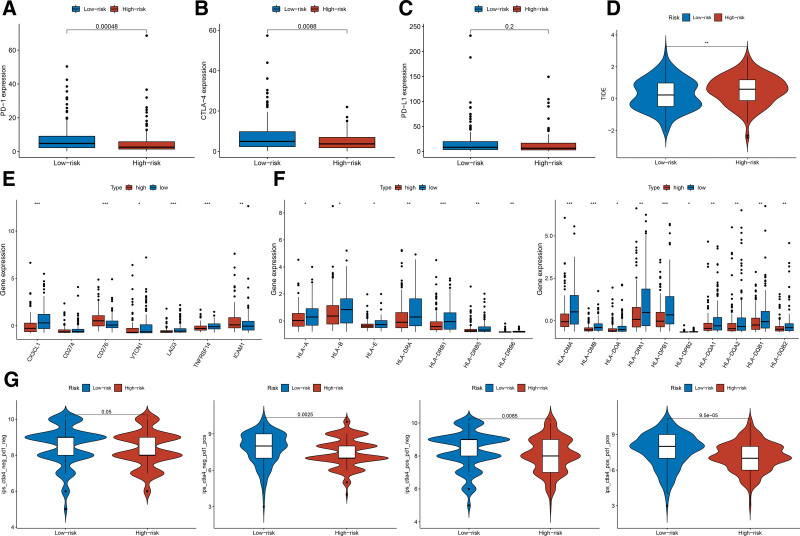
The sensitivity analysis of immunotherapy in the prognostic risk signature. (A) Boxplots of the expression of PD-1 in high-risk and low-risk groups. (B) Boxplots of the expression of CTLA-4 in high-risk and low-risk groups. (C) Boxplots of the expression of PD-l1 in high-risk and low-risk groups. (D) The difference of TIDE score in high-risk and low-risk groups. The asterisks represented the statistical *P* value (**P < .01). (E) The expression of immune checkpoint genes in high-risk and low-risk groups. The asterisks represented the statistical *P* value (*P < .05; **P < .01; ***P < .001). (F) The expression of HLA genes in high-risk and low-risk groups. The asterisks represented the statistical *P* value (*P < .05; **P < .01; ***P < .001). (G) The difference of IPS score in high-risk and low-risk groups. HLA = human leukocyte antigen, IPS = immunophenoscore, TIDE = tumor immune dysfunction and exclusion.

Recent studies have demonstrated that immune checkpoint inhibitors (ICB) are particularly effective in the treatment of CC. To evaluate the feasibility of the prognostic risk signature for predicting responses to immunotherapy, we compared the correlation between immune checkpoints and risk scores. Our findings revealed that *PD-1*, *CTLA-4*, *VTCN1*, *LAG3*, and *TNFRSF14* were highly expressed in the low-risk group (Fig. 5A–C, E). Additionally, *HLA-A*, *HLA-B*, *HLA-E*, and other *HLA* genes exhibited elevated expression levels in the low-risk group (Fig. 5F). TIDE score predictions indicated that the low-risk group had lower scores, suggesting a decreased likelihood of immune escape and a better response to immunotherapy (Fig. 5D). Furthermore, IPS analysis revealed higher expression levels in the CTLA-4^−^PD-1^+^, CTLA-4^+^PD-1^−^, and CTLA-4^+^PD-1^+^ subgroups within the low-risk group, indicating potential benefits from immunotherapy. These findings are consistent with the predicted results of the TIDE score (Fig. 5G). Therefore, the prognostic risk signature may serve as a reliable predictor of immunotherapy outcomes in patients with CC.

**Figure 6.**
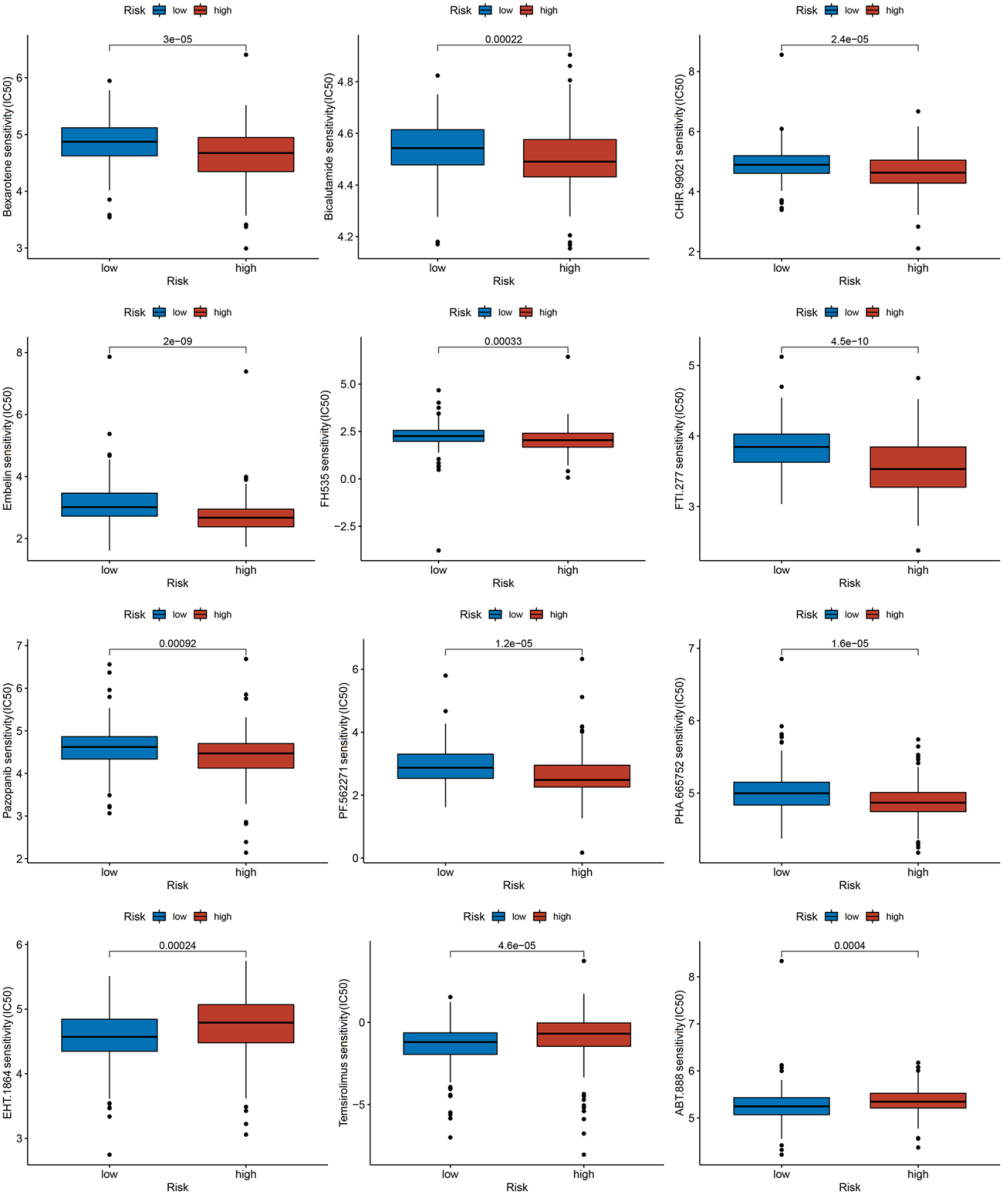
The sensitivity analysis of chemotherapy in the prognostic risk signature.

Moreover, we investigated the potential of the prognostic risk signature in predicting the efficacy of chemotherapy. The high-risk group was increased sensitive to bexarotene, bicalutamide, CHIR.99021, embelin, FH535, FTI.277, pazopanib, PF.562271, and PHA.665752, whereas the low-risk group benefited from EHT.1864, temsirolimus, and ABT.888 (Fig. 6).

### 3.9. Validation of disulfidptosis-related lncRNAs

To further validate the accuracy of the prognostic risk signature, we evaluated the expression levels of 9 disulfidptosis-related lncRNAs in 304 CC samples and 3 normal cervical samples from the TCGA dataset. The results indicated that AP001037.1, AL139351.3, ILF3-DT, AC119427.1, and AC004540.2 had lower expression levels in CC compared to normal cervical samples, while AC119427.1, PPP1R14B-AS1, AL021707.6, and RUSC1-AS1 demonstrated higher expression levels in CC (Fig. S5A, Supplemental Digital Content, https://links.lww.com/MD/Q658). Subsequently, we selected 3 disulfidptosis-related lncRNAs (ILF3-DT, PPP1R14B-AS1, and RUSC1-AS1) for qPCR detection in 5 pairs of CC and paired adjacent normal tissues. The qPCR results indicated that the expression levels of ILF3-DT and RUSC1-AS1 were consistent with our previous bioinformatics analysis, whereas the expression of PPP1R14B-AS1 was divergent (Fig. S5B, Supplemental Digital Content, https://links.lww.com/MD/Q658).

## 4. Discussion

Disulfidptosis is a novel form of RCD induced by disulfide stress, distinct from apoptosis, pyroptosis, ferroptosis, and cuproptosis.^[16]^ This mechanism has the potential to serve as a new therapeutic strategy for targeting tumor cells. In this study, we compared the expression levels of 10 disulfidptosis-related genes reported in the literature between CC and normal cervical samples. Our analysis revealed that *GYS1*, *NDUFA11*, *RPN1*, and *SLC7A1*1 were significantly overexpressed in CC and high expression levels of LRPPRC, NCKAP1, SLC3A2, and SLC7A11 was associated with poor prognosis. Notably, *SLC7A11* is highly expressed in lung, liver, pancreatic, and breast cancers, suggesting its potential role in tumor proliferation, metastasis, and chemotherapy resistance.^[17]^ Similarly, LRPPRC is involved in mitochondrial function and is upregulated in gastric cancer, hepatocellular carcinoma, and lung adenocarcinoma.^[18–20]^ the roles of disulfidptosis-related genes in CC remain poorly understood. A previous study reported that circEPSTI1 regulates SLC7A11 expression via sponging miR-375, miR-409-3p, and miR-515-5p in CC.^[21]^

LncRNAs have been identified as key regulators of gene expression in tumor cells, modulating oncogenic or tumor-suppressive functions through epigenetic mechanisms.^[22]^ Numerous studies have focused on screening lncRNAs associated with cell death across various tumor types and developing risk signatures to predict tumor prognosis. For instance, Jiang et al^[23]^ identified anoikis-related lncRNAs and developed prognostic signatures that utilized these lncRNAs to predict the efficacy of immunotherapy and the prognosis of patients with pancreatic adenocarcinoma. Similarly, Cheng et al^[24]^ developed a prognostic signature for prostate cancer based on 6 cuproptosis-related lncRNAs, demonstrating significant associations between the signature and immune cell infiltration, immune checkpoint expression, TMB, microsatellite instability, and chemosensitivity. Wu et al^[25]^ constructed a 4-ferroptosis-related-lncRNA signature for colon cancer and validated its prognostic value using Kaplan–Meier and ROC analyses, suggesting its potential as a novel biomarker. However, studies on prognostic risk signatures derived from lncRNAs specifically associated with disulfidptosis remains limited. In this study, we constructed a prognostic risk signature of 9 disulfidptosis-related lncRNAs using LASSO and Cox regression analyses. The Kaplan–Meier curve, ROC curve, and C-index demonstrated that the signature exhibited good sensitivity and specificity. Among these 9 lncRNAs, AP001037.1, AL139351.3, ILF3-DT, AC119427.1, and AC004540.2 were downregulated in CC, whereas AC119427.1, PPP1R14B-AS1, AL021707.6, and RUSC1-AS1 were upregulated. Notably, these lncRNAs, with the exception of RUSC1-AS1,^[26,27]^ have rarely been reported in CC or other tumors. Previous studies have indicated that ILF3-DT is associated with autophagy^[28]^ and both AC119427.1 and AC004540.2 have been linked to fatty acid metabolism.^[29]^ Nevertheless, the molecular mechanisms through which these 9 disulfidptosis-related lncRNAs contribute to the pathogenesis and progression of CC remain largely unknown.

The tumor immune microenvironment plays a crucial role in regulating tumor development, infiltration, and metastasis. It is primarily composed of infiltrating immune cells, chemokines, and cytokines.^[30,31]^ In recent years, immunotherapy, particularly ICB, has revolutionized cancer treatment. According to the 2022 NCCN guidelines, pembrolizumab in combination with chemotherapy and bevacizumab is recommended as the first-line treatment for patients with recurrent or metastatic CC positive for PD-1/PD-L1.^[32,33]^ However, the response rate to immunotherapy remains relatively low, ranging from 10% to 58%, which may be partly attributed to the tumor immune escape mechanism driven by the heterogeneous tumor immune microenvironment.^[34,35]^ In this study, we utilized the CIBERSORT and ESTIMATE algorithms to analyze ImmuneScore and ESTIMATEScore, revealing that the low-risk group exhibited higher scores than the high-risk group. In the low-risk group, CD8^+^ T cells and activated CD4^+^ memory T cells were significantly enriched. Additionally, scores for HLA, immune checkpoints, cytokines, and activated immune promotion were higher in the low-risk group. Both TIDE score and IPS indicated that the low-risk group represents an immune-inflamed (“hot tumor”) phenotype, which is associated with increased sensitivity to ICB therapy.^[36]^ In contrast, the high-risk group was characterized as an immune-desert (“cold tumor”) phenotype, demonstrating lower ICB sensitivity.^[37]^

We also compared the potential response to chemotherapy between high-risk and low-risk groups. Patients in the high-risk group showed increased sensitivity to pazopanib, CHIR-99021, and FH535, whereas those in the low-risk group were more likely to benefit from temsirolimus, EHT-1864, and ABT-888. Pazopanib, a tyrosine kinase inhibitor, targets the vascular endothelial growth factor receptor, platelet-derived growth factor receptor, fibroblast growth factor receptor, and cytokine receptor.^[38]^ A Phase II clinical trial (NCT00430781) evaluating pazopanib alone or in combination with lapatinib in patients with recurrent or metastatic CC reported a significant prolongation of PFS with a manageable safety profile.^[39]^ Temsirolimus, an mTOR inhibitor, effectively suppresses the PI3K/Akt/mTOR signaling pathway, which is abnormally activated in various tumor cells, thereby inhibiting their malignant behavior.^[40]^ Phase II trial data indicated that temsirolimus monotherapy led to partial response in 3.0% of patients with recurrent, advanced, or metastatic CC, stable disease in 57.6%, and a 6-month PFS rate of 28%.^[41]^ ABT.888, also known as veliparib, is a novel highly selective oral inhibitor targeting poly-ADP-ribosepolymerase.^[42]^ A Phase I trial (NCT01281852) evaluating veliparib combined with paclitaxel and cisplatin in recurrent or metastatic CC reported an objective response rate of 60%, supporting the safety and feasibility of this regimen.^[43]^ In contrast, FH535, CHIR-99021, and EHT-1864 have demonstrated antitumor effects only at the cellular or animal level, and have not yet advanced to clinical trials. Further investigations are warranted to determine their potential therapeutic utility.

This study has several limitations. We exclusively utilized the TCGA database to construct and validate the prognostic risk signature, without conducting in vitro experiments to further confirm the influence of disulfidptosis-related lncRNAs on the occurrence and progression of CC. Future work should include external validation using independent datasets and experimental functional studies to enhance biological interpretation. Additionally, we aim to collect a substantial amount of clinical data in future research to validate the accuracy and applicability of this signature.

## 5. Conclusions

In conclusion, we constructed a prognostic risk signature based on 9 disulfidptosis-related lncRNAs in patients with CC. This signature may assist in predicting prognosis, enhancing our understanding of the tumor immune microenvironment, and providing guidance for immunization and targeted therapies, thereby improving the precision of treatment and clinical outcomes for patients with CC.

## Acknowledgments

We thank all the researchers involved in merging and submitting TCGA database data, which may provide convenience and possibility for large-scale cancer research.

## Author contributions

**Conceptualization:** Hu Zhao, Yingmei Wang.

**Data curation:** Lu Wang, Rui Li.

**Funding acquisition:** Hu Zhao, Yilin Guo.

**Methodology:** Yilin Guo.

**Project administration:** Yingmei Wang.

**Software:** Yilin Guo, Lu Wang.

**Validation:** Hu Zhao, Rui Li.

**Writing – original draft:** Hu Zhao, Yilin Guo.

**Writing – review & editing:** Lu Wang, Yingmei Wang.

## Supplementary Material


