# Supplementary material for: A novel disulfidptosis-related lncRNA signature to predict prognosis and immune response of cervical cancer
Source: Medicine (Baltimore). 2025 Nov 21;104(47):e46023. doi: 10.1097/MD.0000000000046023 (PMC12643716; doi:10.1097/MD.0000000000046023)

Supplementary Figure 1 A: Risk score distribution of CC patients in training cohort. B: Survival status distribution of CC patients in training cohort. C: The heatmap of the expression levels of 9 disulfidptosis-related lncRNAs in training cohort. D: Risk score distribution of CC patients in validation cohort. E: Survival status distribution of CC patients in validation cohort. F: The heatmap of the expression levels of 9 disulfidptosis-related lncRNAs in validation cohort. G: ROC curve of 1-, 3-, and 5-years in training cohort. H: ROC curve of risk score and clinicopathological features in training cohort. I: ROC curve of 1-, 3-, and 5-years in validation cohort. J: ROC curve of risk score and clinicopathological features in validation cohort.

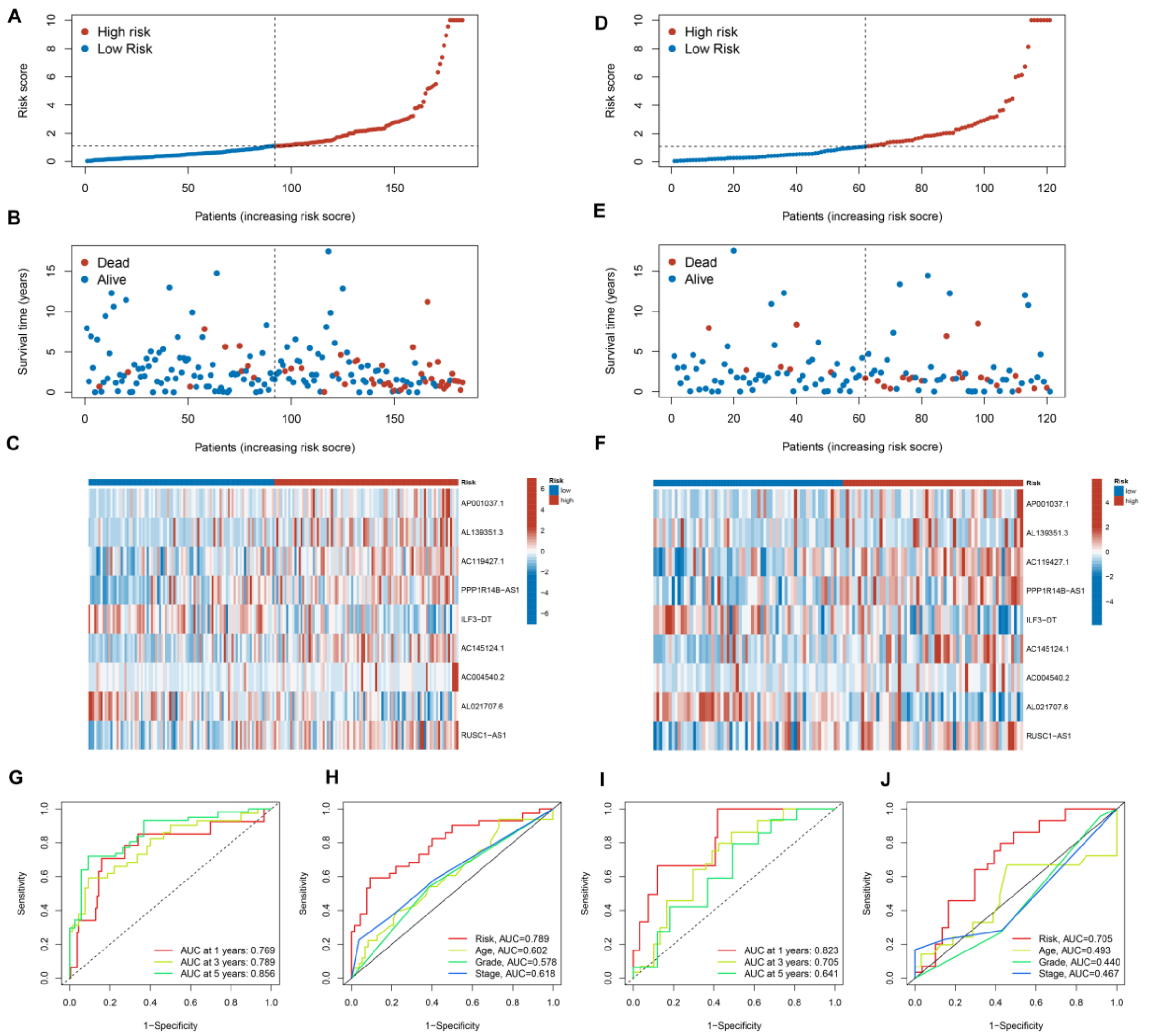

Supplementary Figure 2 Establishment of a nomogram and subgroup analysis A: Nomogram of risk score and clinicopathological features. B: Calibration curve for 1-, 3-, and 5-years survival of the nomogram. C: Kaplan–Meier survival curves of age≤65 years in high-risk and low-risk groups. D: Kaplan–Meier survival curves of G1-G2 in high-risk and low-risk groups. E: Kaplan–Meier survival curves of age>65 years in high-risk and low-risk groups. F: Kaplan–Meier survival curves of G3 in high-risk and low-risk groups. G: Kaplan–Meier survival curves of stage I-II in high-risk and low-risk groups. H: Kaplan–Meier survival curves of stage III-IV in high-risk and low-risk groups.

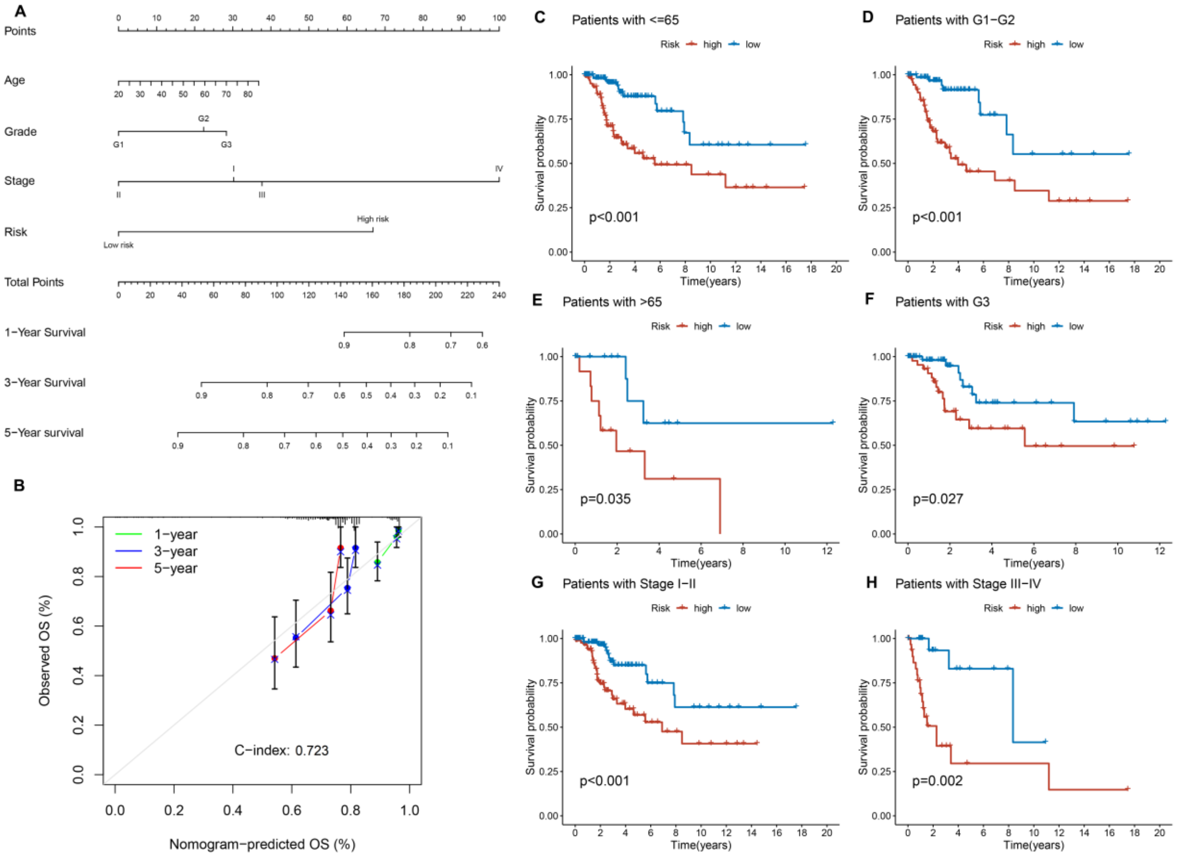

Supplementary Figure 3 A: PCA analysis on the total gene expression profiles. B: PCA analysis on the disalfidposis-related gene expression profiles. C: PCA analysis on the disalfidposis-related lncRNAs expression profiles. D: PCA analysis on the 9 disulfidptosis-related lncRNAs expression profiles of the risk prognosis signature. E: GO enrichment analysis of DEGs in the high-risk and low-risk groups. F: GSVA analysis in high-risk and low-risk groups. G: GSEA analysis in high-risk and low-risk groups.

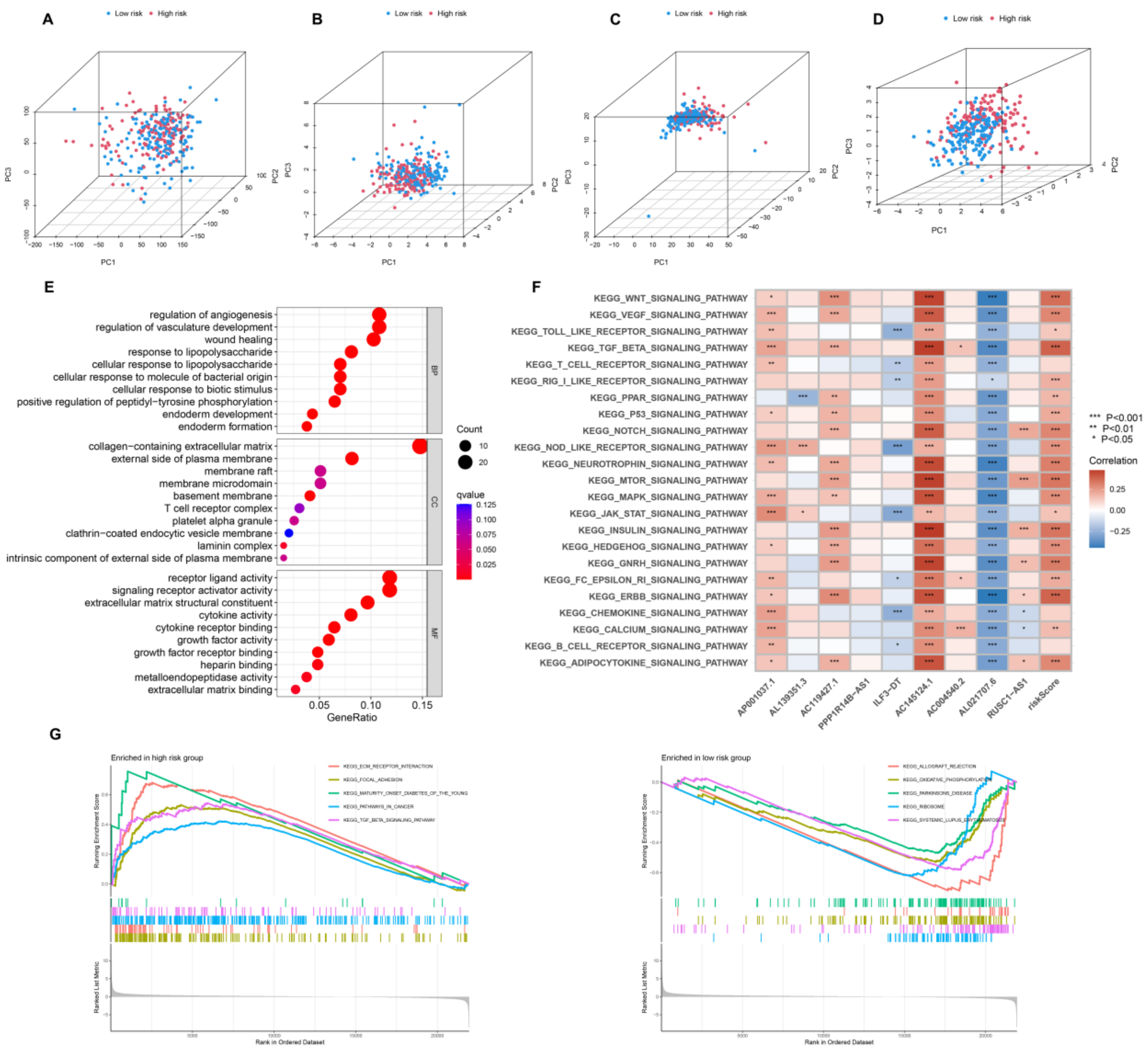

Supplementary Figure 4 A: Waterfall plot of the top 15 mutation genes in the low-risk group. B: Waterfall plot of the top 15 mutation genes in the high-risk group. C: Boxplots of TMB in high-risk and low-risk groups. D: Kaplan–Meier survival curves of TMB in CC. E: Kaplan–Meier survival curves of TMB and risk score in CC. F: The expression of proliferation-related genes, DNA repair-related genes, and angiogenesis-related genes in high-risk and low-risk groups. The asterisks represented the statistical p value (*P < 0.05; **P < 0.01; ***P < 0.001).

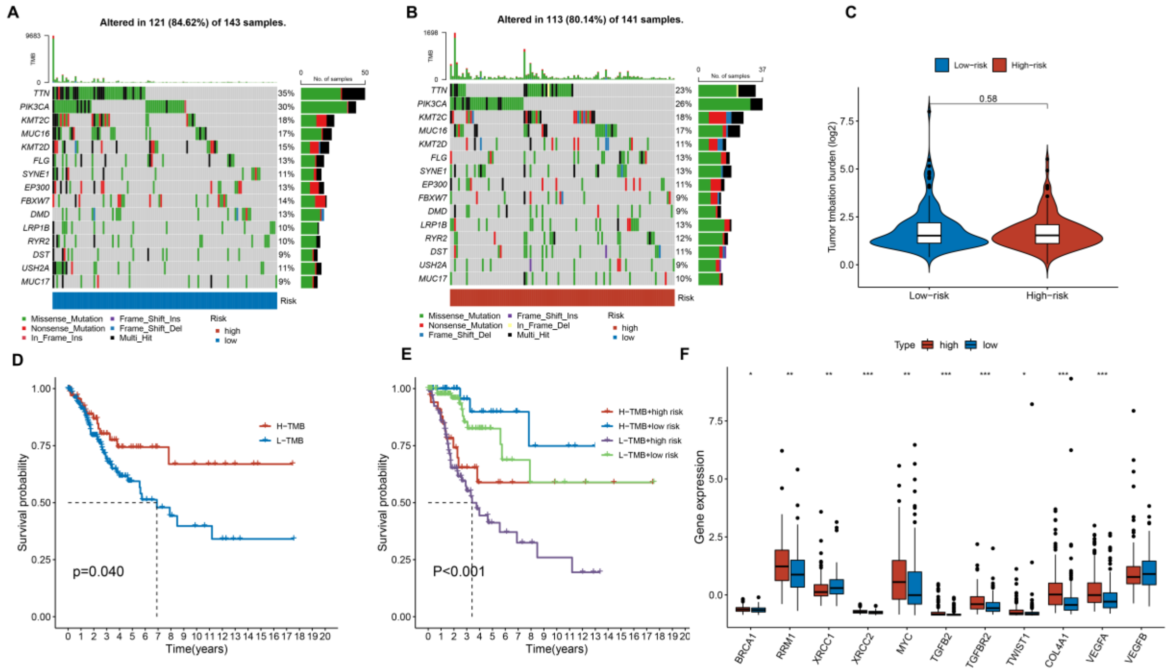

Supplementary Figure 5 Validation of the disulfidptosis-related lncRNAs expression level in the prognostic risk signature. A: The 10 disulfidptosis-related lncRNAs expression level of the prognostic risk signature in TCGA dataset. The asterisks represented the statistical p value (*P < 0.05; **P < 0.01). B: The ILF3-DT, PPP1R14B-AS1, and RUSC1-AS1 expression level in 5 pairs of CC and paired adjacent normal tissues via qPCR. The asterisks represented the statistical p value (*P < 0.05; ***P < 0.001).

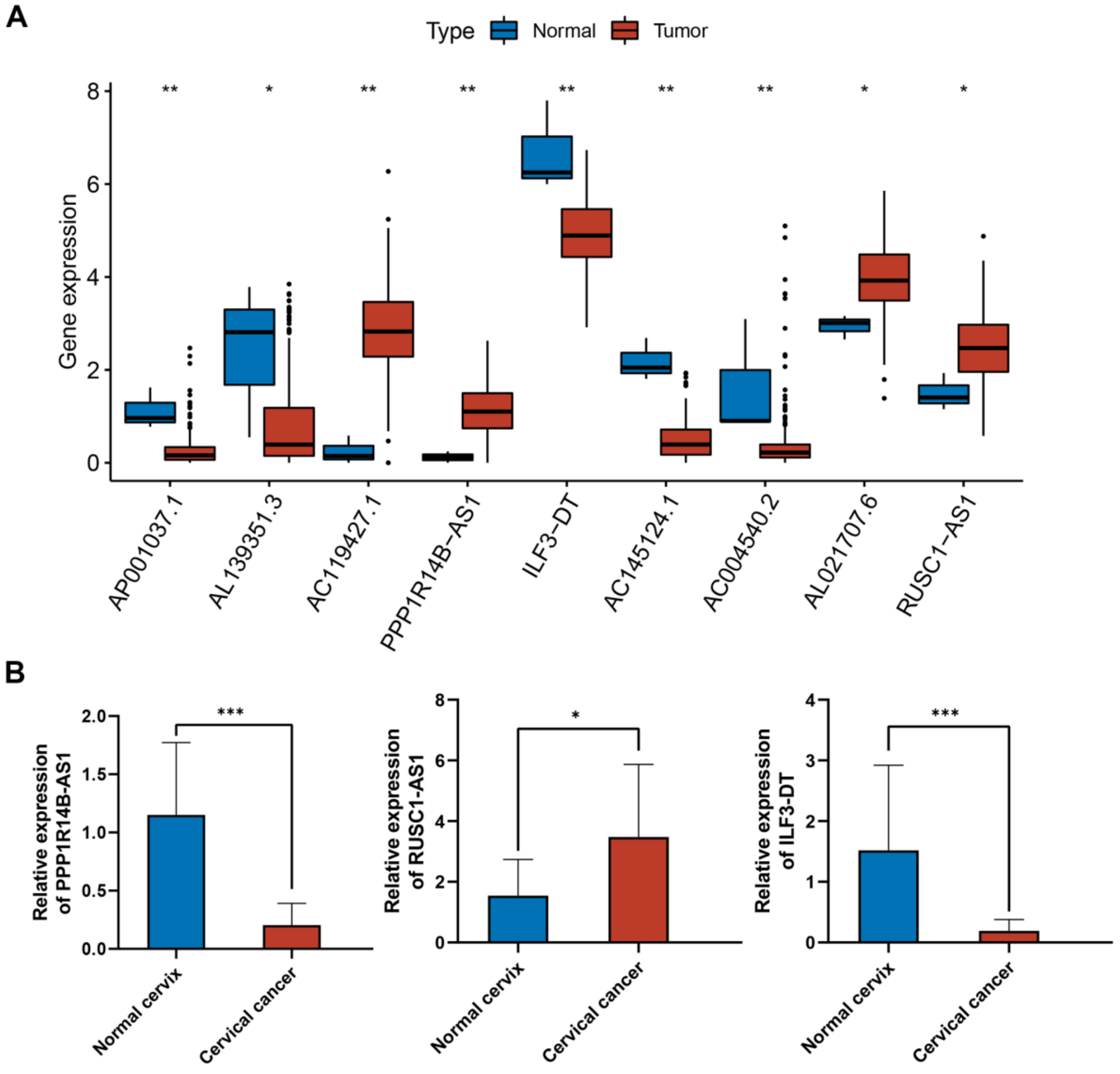

Supplement: Supplementary file 1 [file medi-104-e46023-s001.pdf]
